# In-field hyperspectral imaging dataset of Manzanilla and Gordal olive varieties throughout the season

**DOI:** 10.1016/j.dib.2022.108812

**Published:** 2022-12-07

**Authors:** Samuel Domínguez-Cid, Julio Barbancho, Diego F. Larios, F.J. Molina, Ariel Gómez, C. León

**Affiliations:** Department of Electronic Technology, University of Seville, Seville, Spain

**Keywords:** Olive, Hyperspectral imaging, Agrotech, Precision agriculture

## Abstract

Because spectral technology has exhibited benefits in food-related applications, an increasing amount of effort is being dedicated to develop new food-related spectral technologies. In recent years, the use of remote sensing or unmanned aerial vehicles for precision agriculture has increased. As spectral technology continues to improve, portable spectral devices become available in the market, offering the possibility of realising in-field monitoring. This study demonstrates hyperspectral imaging and spectral olive signatures of the Manzanilla and Gordal cultivars analysed throughout the table-olive season from May to September. The data were acquired using an in-field technique and sampled via a non-destructive approach. The olives were monitored periodically during the season using a hyperspectral camera. A white reference was used to normalise the illumination variability in the spectra. The acquired data were saved in files named raw, normalised, and processed data. The normalised data were calculated by the sensor by correcting the white and black levels using the acquired reflectance values. The olive spectral signature of the images is saved in the processed data files. The images were labelled and processed using an algorithm to retrieve the olive spectral signatures. The results were stored as a chart with 204 columns and ‘n’ rows. Each row represents the pixel of an olive in the image, and the columns contain the reflectance information at that specific band. These data provide information about two olive cultivars during the season, which can be used for various research purposes. Statistical and artificial intelligence approaches correlate spectral signatures with olive characteristics such as growth level, organoleptic properties, or even cultivar classification.

**Specifications Table**

**Table:** 

Subject	*Agriculture Engineering*
Specific subject area	*Agrotech, Hyperspectral imaging, precision agriculture, pattern recognition, Cyber–Physical system integrations.*
Type of data	*Excel file* *Hyperspectral images in ENVI format.*
How the data were acquired	*In-field hyperspectral images (HSIs) were acquired using a hyperspectral camera from manufacturer SPECIM, Finland. The model is SPECIM IQ.*
Data format	Raw and normalised HSIs are in ENVI ‘.hdr’ format. The processed data are stored as ‘.xlsx’ format.
Description of data collection	*An in-field hyperspectral image sensor was used for data acquisition.* *An olive spectral signature file was obtained by processing the HSI.* *Data recording was performed during the table-olive season from May to September.*
Data source location	*Data acquisition was carried out in an olive field in a city on the north-west side of Seville in the south of Spain.* • *City/Town/Region: Espartinas, Seville province* • *Country: Spain* • *Latitude and longitude: 37.394327, -6.121881*
Data accessibility	*Repository name: Mendeley Data* *Data identification number:*http://dx.doi.org/10.17632/8xvhcsdvst.1 *Direct URL to data:*https://data.mendeley.com/datasets/8xvhcsdvst/1

## Value of the Data

• In smart agro applications, there are technological approaches that use artificial intelligence or traditional statistical methods such as ANOVA or PLS [1], [2], [3], which require the use of data. In this regard, data are essential for both artificial intelligence and stochastic approaches. There are hyperspectral developments in the remote sensing context using satellite mission data such as those from MODIS, VIIRS, or SENTINEL [4,5] although the in-field data available are limited. The proposed approach enables new methodologies and cross-validation of models used in remote sensing applications. Moreover, in-field information is detailed in terms of resolution, resulting in new information that cannot be acquired by satellite imaging.
• The data were gathered in the field throughout the season for two specific table-olive cultivars that have a PGI [6]. These PGI cultivars are Gordal and Manzanilla in the province of Seville [7]. The dataset comprises hyperspectral images, RGB images, and spectral data in the range of 400–1000 nm. The data were processed to obtain the spectral signatures of the olives in the images. This can be a starting point for researchers who wish to analyse olive cultivars using in-field data. Laboratories have analysed olives using spectral techniques to extract their chemical composition and correlate the spectral signature with organoleptic properties [8].
• These data are a high-value source of information for researchers and developers to create new methodologies for generating knowledge from raw data. Therefore, data can be used to develop new algorithms regarding general/specific purpose applications, enhance normalisation techniques of in situ acquisition, or even develop new techniques for the segmentation or classification of Hyperspectral Images (HSIs).
• The data published here can be used to compare acquisition methodologies, as a source for algorithms, or even to complement techniques with an in-field approach. A large number of remote sensing datasets are provided by governmental agencies, although the variety in field data is limited [9], [10], [11]. High-value development may be addressed to analyse olive properties or characteristics, study the correlation between olive signature variations during the growing season, compare different cultivars, or even analyse the variations with weather conditions.

## 1 Objective

This dataset contains in-field data of the spectral signature of olives at the pixel level, processed and obtained from the images acquired. In addition, the dataset contains the HSIs acquired during the table-olive season from May to September, which are the images processed to obtain the spectral profiles. The data are a source of valuable information to validate models from different acquisition methodologies, or they may be used to correlate the chemical properties of olives, as performed in laboratories. In this regard, a novel, non-invasive in-field approach is developed to acquire data.

## 2 Data Description

**Fig. 1:**
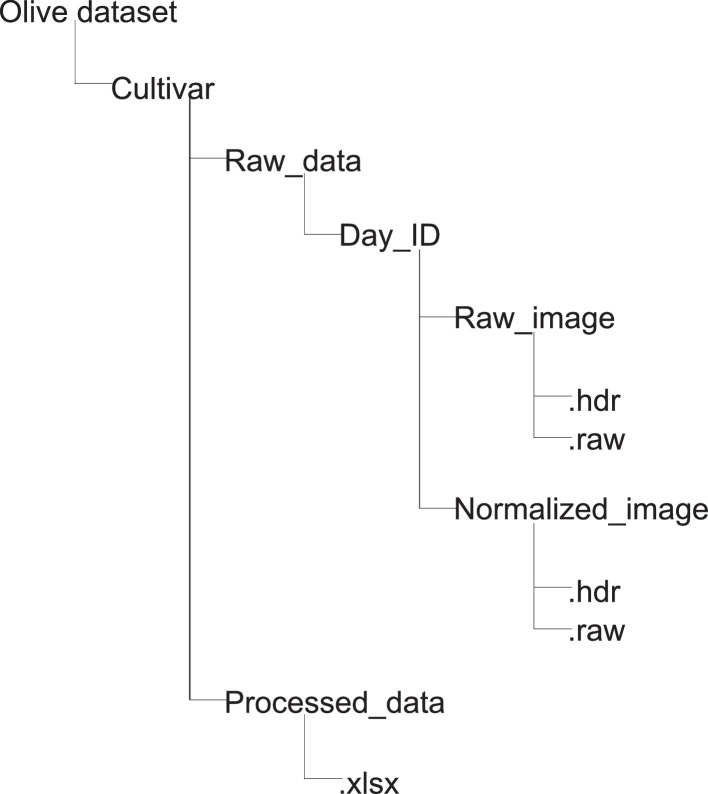
Organisation structure of the hyperspectral image dataset.

The data are stored in a common folder called ‘Olive_dataset’. In this folder, the information is divided into two main folders, one for each of the monitored cultivars, ‘Gordal’ and ‘Manzanilla’. In these folders, the structure is identical for each cultivar, as shown in Fig. 1. Therefore, each cultivar folder contains two deeper folders inside the ‘Day_timestamp’ folder, ‘raw_data’ and ‘processed_data’, for organising the files. The folder ‘processed_data’ contains xlsx files. These files are named using the structure <location>_<YYYY>-<MM>-<DD>-<cultivar>-<ID>; the tags are replaced as follows:

• <location>: Location from where the data were acquired.
• <YYYY>: Year in which the data were acquired.
• <MM>: Month when data were acquired.
• <DD>: Day when the data were acquired.
• <ID>: Identification number for samples collected on a particular day.

Each row in the file represents a labelled pixel in the HSI. The columns represent the bands or wavelengths for which the sensor can obtain the reflectance. For every pixel, 204 different bands were used ranging from 400–1000 nm. The reflectance value of the i^th^ pixel at j^th^ wavelength is the value at position (i, j), an example of which is shown in Table 1.

**Table 1:** Example of the reflectance values of the processed data stored in an excel sheet (xlsx).

Band ID	1	2	3	…	204
Wavelength in nanometres	397.32	400.2	403.09	…	1003.58
Reflectance pixel 1	0.119047619	0.078125	0.086419754	…	0.600000024
Reflectance pixel 2	0.095238097	0.09375	0.098765433	…	0.600000024
Reflectance pixel 3	0.095238097	0.123076923	0.074074075	…	0.666666687
Reflectance pixel 4	0.119047619	0.107692309	0.086419754	…	0.600000024
Reflectance pixel 5	0.119047619	0.107692309	0.098765433	…	0.600000024
Reflectance pixel 6	0.119047619	0.123076923	0.098765433	…	0.533333361

Inside ‘raw data’, there are several additional folders that follow the previous naming structure. Every folder contains information about a hyperspectral image. Additionally, the sensor automatically applies white normalisation, which is performed using a white reference placed at the scene. This information can be addressed by examining the folder. Two additional folders contained raw and normalised files of the hyperspectral images. In any of these, the files are saved with an extension ‘.hdr’, ‘.raw’, or ‘.dat’. Following the ENVI file format, the ‘.hdr’ files contain the hyperspectral headers and metadata of the HSI, and ‘.raw’ files contain the hypercube itself. As previously described, the names of the files were saved as <location>_<YYYY>-<MM>-<DD>-<cultivar>-<ID>.<extension>, with .<extension> being ‘.hdr’, ‘.raw’, or ‘. dat’ format. In the normalised folder, the name is preceded by the text ‘REFLECTANCE’ to indicate that the hyperspectral image has been processed by a sensor with white and black reflectances.

## 3 Experimental Design, Materials, and Methods

The sensor used for acquiring the images was the hyperspectral camera SPECIM IQ, from SPECIM, SPECTRAL IMAGING LTD., Oulu, Finland (+358). The model used for sampling comprises an RGB and hyperspectral camera. The camera captures RGB and hyperspectral images simultaneously, providing the possibility of observing the analysed scenario. The hyperspectral image is 512 × 512 in size with 204 bands (each having 7 nm resolution) spanning from 400–1000 nm of the spectra. A white and black plate was used as the reference to normalise the white and black reflectance values. It had a rectangular shape of size 5 × 5 cm and was made of white polytetrafluoroethylene (PTFE) with a black framework. The material used in the plate exhibited a stable reflectance signature along the working wavelength of the hyperspectral sensor [12]. The use of a reference is critical for reducing the illumination variabilities between the samples.

**Fig. 2:**
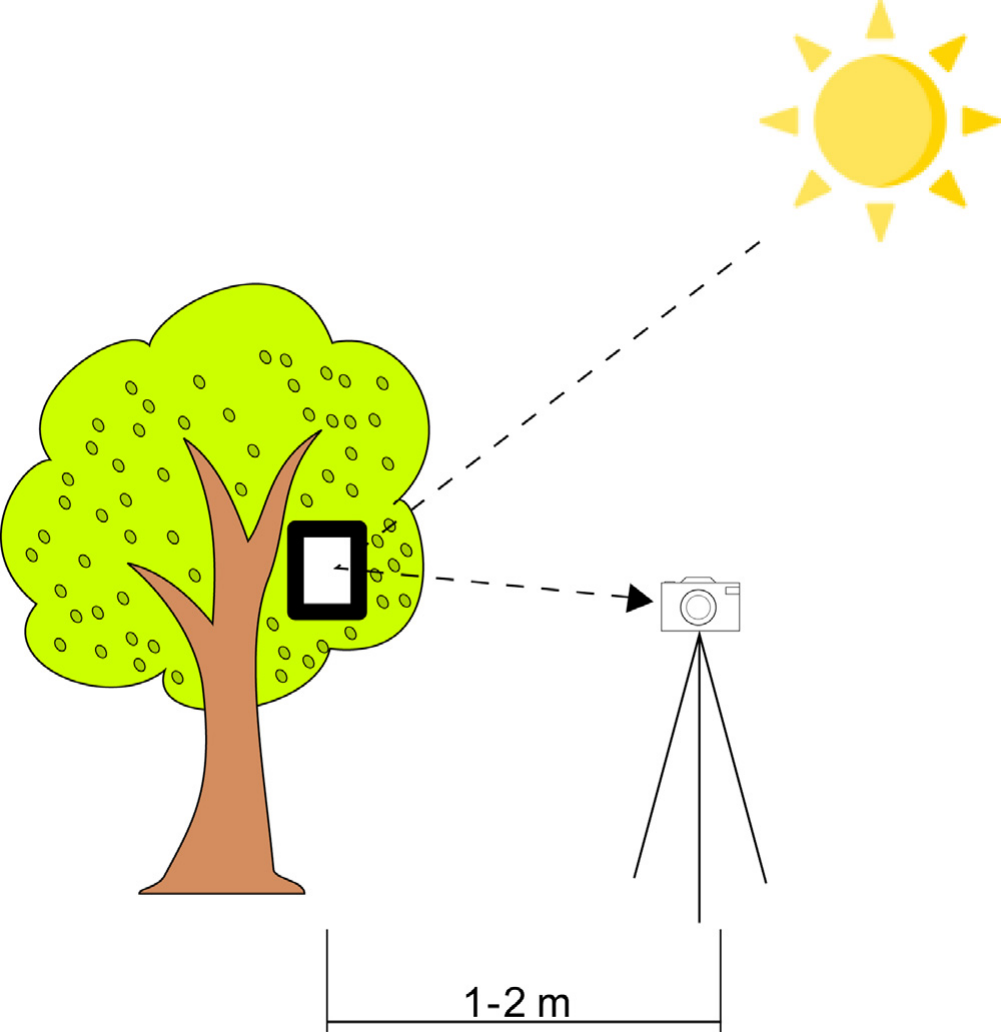
In-field acquisition set-up diagram.

The experiment was performed using an in-field acquisition hyperspectral imagery methodology. The sensor was placed in the analysed area using a tripod to ensure stability during image acquisition, as shown in Fig. 2. Depending on the selected exposure time, the image was captured in a slow or fast manner. This exposure time depended on the illumination conditions; therefore, it changed with the weather. Regarding illumination variations, the images were captured from 9:00 to 10:00 in the morning from the end of May to the first few days of October. Additionally, the white reference was set in all images to normalise the extreme maximum and minimum values of white and black. The reference was placed in the scene in direct sunlight to saturate with the maximum values. The sensor calculated the normalisation with the white reference values; hence, the normalised hyperspectral image had its reflectance values processed using the following equation:

$$R_{norm}=\frac{R_{raw}-Black}{Black-White}$$

**Fig. 3:**
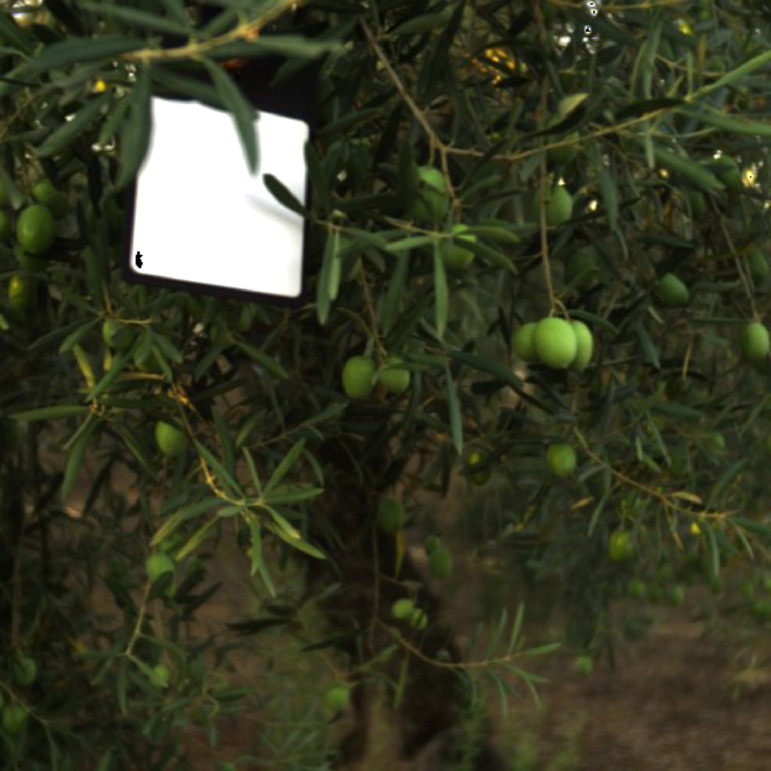
RGB conversion of the hyperspectral normalised image.

**Fig. 4:**
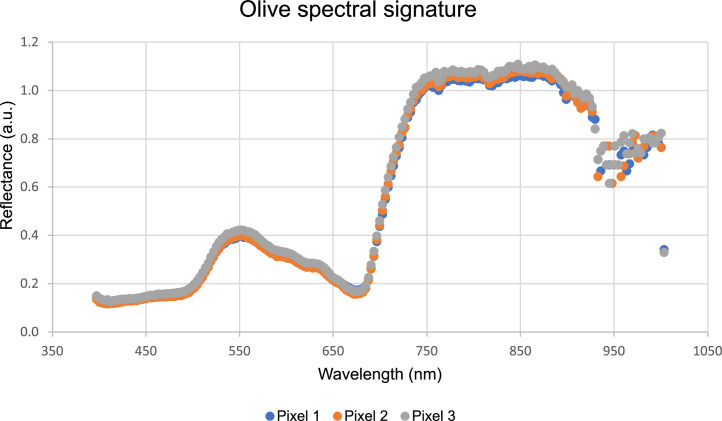
Spectral signature of olives at different pixels.

Two processes were performed to generate the excel files. First, the olives in the images were identified using a graphical representation of the hypercube. To perform this process, the HSI was converted into an RGB image such that a human could identify the olives, as depicted in Fig. 3. The fruits were then labelled in the image using a self-made image processing software similar to labelme [13]. The pixels that belong to the olives were selected using the graphical tools of the software. The selected pixels created a mask file as the output of the software. These images were then processed by an algorithm to extract various regions from the images and obtain their corresponding coordinates. MATLAB and its image processing and computer vision toolbox [14] were used for this development. The areas and centroid coordinates were superposed with the hyperspectral cube, thereby extracting the entire wavelength within the contours of the area. Finally, the signature of each pixel was saved in an excel file. Therefore, the generated file contains olive signatures from 400–1000 nm wavelength. The spectral profile of the olive obtained using this approach is shown in Fig. 4, which plots the reflectance in the wavelength range of the hyperspectral sensor.

## Ethics Statements

No human or animal subjects were used during experimental data collection. Furthermore, the data were not collected from social media platforms.

## CRediT authorship contribution statement

**Samuel Domínguez-Cid:** Funding acquisition, Writing – original draft. **Julio Barbancho:** Funding acquisition, Writing – review & editing. **Diego F. Larios:** Conceptualization, Writing – review & editing. **F.J. Molina:** Conceptualization. **Ariel Gómez:** Writing – review & editing, Funding acquisition. **C. León:** Supervision, Writing – review & editing.

## Declaration of Competing Interest

The authors declare that they have no known competing financial interests or personal relationships that could have influenced the work reported in this paper.

## Data Availability

In-field hyperspectral imaging dataset of Manzanilla and Gordal olive varieties throughout the season (Original data) (Mendeley Data).

## References

[1] Deiana P., Santona M., Dettori S., Culeddu N., Dore A., Molinu M.G. (2019). Multivariate approach to assess the chemical composition of Italian virgin olive oils as a function of variety and harvest period. Food Chem..

[2] Conte P., Difonzo F.C.G., Squeo G., Del Caro L.M.A., Urgeghe P.P., Fadda C., Montinaro A., Piga A. (2019). Change in quality during ripening of olive fruits and related oils extracted from three minor autochthonous Sardinian cultivars. Emir. J. Food Agric..

[3] Reboredo-Rodríguez P., Olmo-García L., Figueiredo-González M., González-Barreiro C., Carrasco-Pancorbo A., Cancho-Grande B. (2020). Effect of olive ripening degree on the antidiabetic potential of biophenols-rich extracts of Brava Gallega virgin olive oils. Food Res. Int..

[4] VIIRS Characterization Support Team (VCST)/ MODIS Adaptive Processing System (MODAPS), “VIIRS/NPP Day/Night Band 6-Min L1B Swath SDR- 750m NRT”, 2017, NASA LANCE MODIS at the MODAPS, doi:10.5067/VIIRS/VNP02DNB_NRT.001.

[5] Donlon C., Berruti B., Buongiorno A., Ferreira M.H., Féménias P., Frerick J., Goryl P., Klein U., Laur H., Mavrocordatos C., Nieke J., Rebhan H., Seitz B., Stroede J., Sciarra R. (2012). The global monitoring for environment and security (GMES) Sentinel-3 mission. Remote Sens. Environ..

[6] Concepción R., García P., Medina E., Brenes M. (2018). The PDO and PGI table Olives of Spain. Eur. J. Lipid Sci. Technol..

[7] Regulatory Council PGI Manzanilla and Gordal from Seville" IGP Aceituna Manzanilla y Gordal de Sevilla", 2020, https://www.igpmanzanillaygordaldesevilla.org/igp/. Last accessed June 1, 2022.

[8] Bengana M., Bakhouche A., Lozano-Sánchez J., Amir Y., Youyou A., Segura-Carretero A., Fernández-Gutiérrez A. (2013). Influence of olive ripeness on chemical properties and phenolic composition of Chemlal extra-virgin olive oil. Food Res. Int..

[9] Gomes L., Nobre T., Sousa A., Rei F., Guiomar N. (2020). Hyperspectral reflectance as a basis to discriminate olive varieties—a tool for sustainable crop management. Sustainability.

[10] Thomas S., Kuska M.T., Bohnenkamp D., Brugger A., Alisaac E., Wahabzada M., Behmann J., Mahlein A.K. (2017). Benefits of hyperspectral imaging for plant disease detection and plant protection: a technical perspective. J. Plant Dis. Prot..

[11] Gold K.M. (2021). Plant disease sensing: studying plant-pathogen interactions at scale. mSystems.

[12] Tsai B.K., Allen D.W., Hanssen L.M., Wilthan B., Zeng J. (2008). A comparison of optical properties between solid PTFE (Teflon) and (low density) sintered PTFE. SPIE Proc., 7065, 9 pp..

[13] K.W. wkentaro, “GitHub - wkentaro/labelme: Image Polygonal Annotation with Python (polygon, rectangle, circle, line, point and image-level flag annotation), 2022, https://github.com/wkentaro/labelme. Last accessed 20 September 2022.

[14] (2021).

